# Effect of e-health intervention on disease management in patients with chronic heart failure: A meta-analysis

**DOI:** 10.3389/fcvm.2022.1053765

**Published:** 2023-02-07

**Authors:** Xueying Ding, Yating Wen, Zimeng Tian, Yaru Wen, Guokun Sun, Rongxing Geng, Wei Fang, Yun Xu

**Affiliations:** ^1^School of Nursing, Weifang Medical University, Weifang, Shandong, China; ^2^College of Integrated Chinese and Western Medicine, Jining Medical University, Jining, Shandong, China; ^3^Department of Joint Surgery, Weifang People's Hospital, Weifang, Shandong, China

**Keywords:** quality of life, self-management, Internet, e-health, CHF

## Abstract

**Objective:**

The aim of this meta-analysis was to assess the impact of e-health interventions on disease management in patients with CHF.

**Methods:**

Six databases including Embase, Web of Science, Scopus, PubMed, Cochrane, and EBSCO were searched by computer. The search time is before May 1, 2022. Odds ratios (OR) were used for binary categorical data and weighted mean differences (WMD) for continuous variables. The 95% confidence intervals (CI) were used to express the effect sizes for both count and measurement data. RevMan 5.4 and Stata 16.0 were employed to complete this meta-analysis.

**Results:**

The study included 22 research studies and 5,149 patients. e-health intervention can effectively reduce all-cause mortality [OR = 0.801, 95%CI: (0.650, 0.987), *P* < 0.05], all-cause hospitalization rate [OR = 0.66, 95%CI: (0.46, 0.95), *P* < 0.05] and heart failure related hospitalization rate [OR = 0.750, 95%CI: (0.632, 0.891), *P* < 0.05]. e-health intervention is also effective in improving the quality of life [WMD = 2.97, 95%CI: (1.54, 4.40), *P* < 0.05] and the self-management ability of patients [WMD = −2.76, 95%CI: (−5.52, −0.11), *P* < 0.05].

**Conclusion:**

e-health interventions can reduce all-cause mortality, all-cause hospitalization, and heart failure-related hospitalization in patients with CHF. Furthermore, it can improve the health-related quality of life of patients.

## Introduction

Chronic Heart Failure (CHF) is a serious, potentially life-threatening condition with an increasing prevalence. As a result, a number of medical and economic problems have been associated with it. Worldwide, the incidence of heart failure is predicted to rise by 46% by 2030 due to an aging population (1). In spite of advancements in heart failure treatment, mortality and health-related quality of life remain unimproved. Also, the cost of disease management for CHF patients is ~1–2% of total healthcare costs and is mainly associated with repeated hospitalizations (2). Yet, multidisciplinary health care management has been effective in reducing CHF-related mortality and hospitalizations. Because of geographical barriers, and socioeconomic reasons, not all patients are able to participate in these treatments (3). Consequently, digital health interventions have become increasingly critical in recent years, particularly during the ongoing COVID-19 pandemic (4). Patients with CHF and other chronic diseases are evolving from the traditional face-to-face follow-up model toward a proactive, real-time technology model that helps patients self-manage (5).

In the healthcare sector, the use of e-health and m-health tools is increasing. e-health include any type of electronic system used in medical practice to monitor or improve their health status (6). While e-health typically involves computer-based online or offline telemedicine, mHealth refers to cell phone applications. These services can stimulate positive changes in health behavior. They can help patients live a healthier lifestyle, or support the diagnosis and treatment of disease by remotely monitoring and managing patients with CHF when needed. The goal of managing CHF is to enhance the patient's quality of life and minimize the risk of mortality. This requires close monitoring of vital signs and an effective working relationship between the patient and the health care professional (7).

Overall, the available evidence suggests that e-health interventions have the potential to improve health outcomes in patients with CHF, but the extent of the impact is uncertain owing to the mixed outcomes reported in the available systematic reviews. The literature already contains several systematic reviews on e-health interventions and CHF. Therefore, we can pool evidence from all reviews to report on effect evaluation. We conducted a meta-analysis of randomized controlled trials to assess the evidence for the effectiveness of e-health interventions for the management of CHF.

## Materials and methods

### Literature search

Meta-analysis was conducted according to the Preferred Reporting Items for Systematic Reviews and Meta-analyses (PRISMA) guidelines. The search databases included Scopus, Embase, Web of Science, PubMed, Cochran and EBSCO database. The articles were published after inception and before June 1, 2022, and were related to the impact of electronic interventions on patients with CHF. Our search strategy encompasses a combination of the following keywords: “eHealth” or “e-health” or “e-therapy” or “m-health” or combined with “Chronic heart failure” or “chronic cardiac failure” or “Cardiac Failure” or “Congestive Heart Failure.” In the selection process, only studies involving humans were considered. All databases were searched using similar strategies. We searched PubMed and other databases using “all the fields”, “titles”, and “keywords”.

### Eligibility criteria

#### Types of participants

Inclusion criteria: (1) The study subjects were aged ≥18 years; (2) The study subjects were patients diagnosed with CHF, with a cardiac function class II-III and a left ventricular ejection fraction (LVEF) < 45%; (3) The study subjects were capable of taking care of themselves and had no communication impairment; (4) All literature involved RCTs on the effects of e-health interventions on patients with CHF.

#### Interventions

The experimental group intervention was any form of e-health intervention model: (1) m-Health, in the form of a mobile device-enabled clinical intervention; (2) Telemedicine, which usually entails the use of telephone or electronic technology to facilitate telemedicine or education. The control group intervention was the usual model of care (which did not include any form of e-health).

#### Outcome indicators

Primary outcome indicators: all-cause mortality (total number of deaths at the end of study follow-up), all-cause hospitalization rate (calculated as the proportion of participants who were hospitalized at least once during follow-up), and heart failure-related hospitalization rate (calculated as the proportion of participants who were hospitalized for heart failure at least once during follow-up).

Secondary outcome indicators: health-related quality of life (The primary instrument is the SF-36, and the higher the score the better the quality of life for patients with heart failure. And the Minnesota Malfunctional Heart Quality of Life Scale (MLHFQ), with higher scores indicating poorer quality of life in heart failure patients). Self-care behavior [The primary instrument was the European Heart Failure Self-Care Behavior Scale (EHFSB)].

### Exclusion criteria

Meta-analyses must exclude any study that meets: (1) Literature with incomplete data reporting. (2) Studies with insufficient data to allow interpretation of results. (3) Reviews, *in vitro* studies, case reports, meeting abstracts, and studies related to animal trials.

### Data extraction and management

Two researchers used document management software to remove duplicate studies by importing all filtered titles and literature abstracts after searching the database. Screening of literature that failed to qualify for inclusion and review of full texts to select literature that meets the criterion. Researchers should consult relevant experts or discuss discrepancies with third-party research if discrepancies exist in the literature screening process. When several papers are presented in the same study, only the ones with the most complete data and those that must adhere to the inclusion criteria should be included. All selected literature meeting the inclusion criteria was classified and analyzed through office software: study author, publication year, study design, intervention methods, follow-up time, patient numbers, and patient gender. When some information is lacking from the study, the original author is contacted by phone or email to obtain the relevant data.

### Literature quality assessment

The risk of bias in the selected literature was assessed according to the methods recommended in the Cochrane Handbook for the Systems Review of Interventions 5.1.0. Among the contents assessed are the randomization method, the allocation concealment design, the blinding methods used, the reporting of research results, the existence of other sources of bias, and any selective reporting of research results, etc. Following are the results: “Yes” indicates correct methodology or complete data, indicating a low risk of bias; “unclear” indicates a medium risk of bias; and “No” indicates incorrect methodology or incomplete data, indicating a high risk of bias. Lastly, data was entered into the Revman 5.4 software, and risk of bias assessment plots were exported.

### Statistical analysis

STATA 16.0 version (Stata Corporation, College Station, TX, USA) was utilized to conduct data analyses. Continuous variables are expressed as the weighted mean difference (WMD), while categorical data are expressed as odds ratios (OR). The effect of counting data and the measurement data was expressed using a confidence interval (CI) of 95%. For the heterogeneity test, when statistics for the data *I*^2^ < 50%, the high homogeneity of the research results can be considered, therefore a fixed-effect model is utilized. When the statistics *I*^2^ ≥ 50%, there may be heterogeneity between the results, so a random effect model was used. If there was evidence that a study differed significantly from other studies in terms of methodology or findings, sensitivity analyses were then carried out exclude these studies from the meta-analysis. Sensitivity analyses were performed if there was evidence that a study differed significantly from other studies in terms of methodology or findings. The re-meta-analysis of the data after sequential deletion of individual studies and comparison of the deleted results with the original results was performed. We conducted subgroup analyses of primary outcome indicators to determine the effects of patient age, e-health intervention model, and region. In addition, to assess the side effects of drug treatment in more detail, subgroup analyses were performed according to different adverse events. To examine publication bias, Begg's and Egger's tests were performed; *P* > 0.05 was considered statistically significant unless otherwise stated.

## Results

### Search results

**Figure 1:**
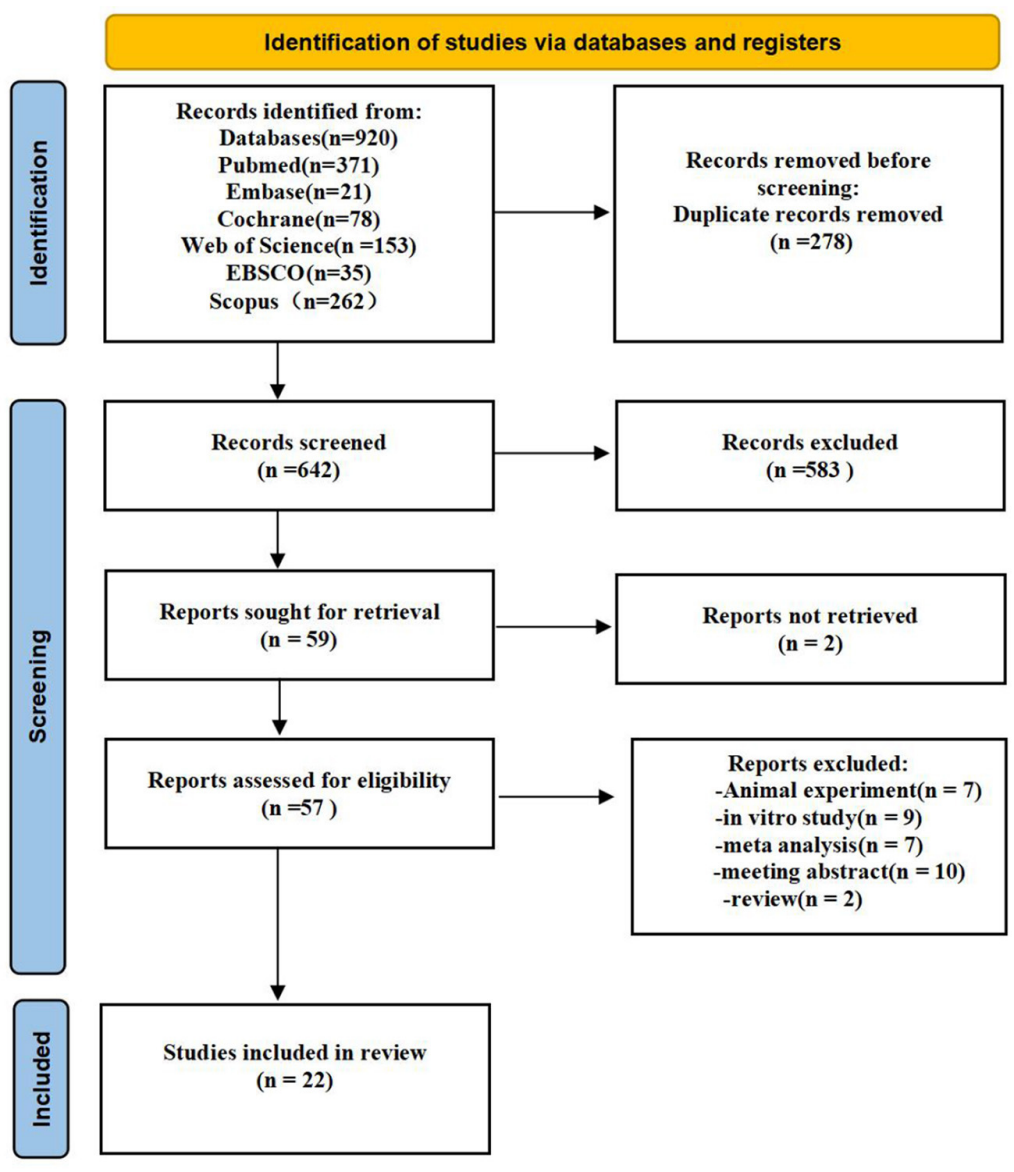
Flow chart of the study selection process.

The search approach is mainly displayed through Supplementary material S1. Figure 1 shows the study selection diagram for the meta-analysis. The PRISMA checklist is shown in the Supplementary Table S2. In the initial search of the database, 920 citations were found (Web of science: 153; Scopus: 262; EBSCO:35; Embase: 21; Cochrane: 78; PubMed: 371;). A total of 642 records remained after removing duplicates, and 583 of them were delegated after titles and abstracts were excluded. Furthermore, the complete texts of 57 articles were read, with 35 being rejected. Finally, 22 studies were included in the meta-analysis.

### Study characteristics and quality assessment

**Figure 2:**
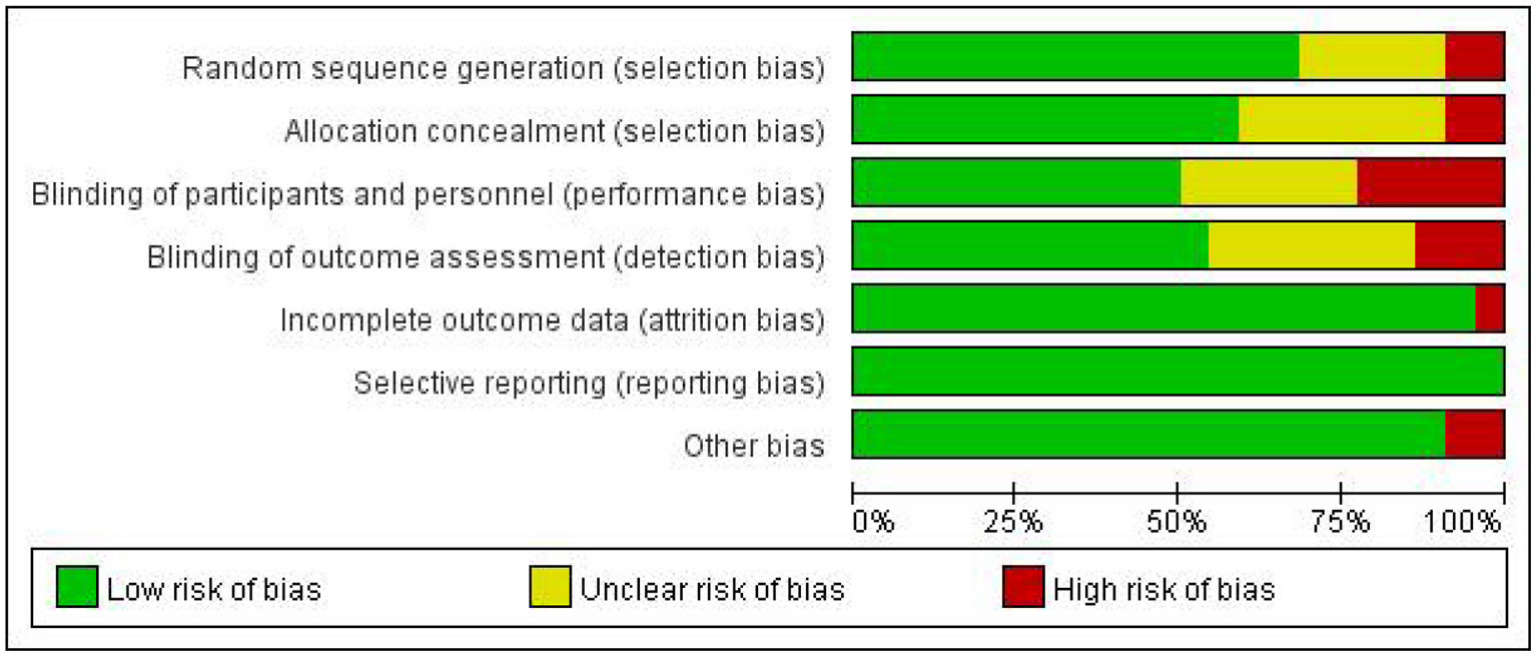
Risk of bias graph.

**Figure 3:**
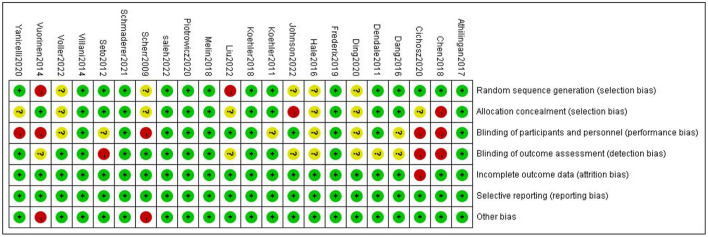
Risk of bias summary.

A total of twenty-two articles were included in this meta-analysis. Table 1 describes the characteristics of the eligible studies. The risk of bias assessment is shown in detail in Figures 2, 3. Fifteen studies' randomization was handled correctly, and thirteen studies dealt with allocation-sequence concealment adequately. Details about participants and personnel blinding were provided in eleven studies, while outcome assessor blinding was reported in twelve more. Each article provided the cause and number of withdrawals and dropouts.

**Table 1:** Characteristics of included studies.

**Included in the study**	**Country**	**Sample size (experimental group / control group)**	**Age (experimental group / control group)**	**Intervention measures of experimental group**	**Intervention measures of control group**	**Outcome measures**
Hale et al. (8)	America	31/14	31.4 ± 11.8/74.4 ± 10.4	Use the drug monitoring system to ensure that the patient takes the medicine according to the regulations.	Usual care	➁
Ding et al. (9)	Australia	31/82	31.5 ± 12.3/70.8 ± 12.4	Use of remote Internet for weight monitoring, structured telephone support, and rehabilitation of patients.	Usual care	➀
Liu2 et al. (10)	China	31/30	31.27 ±7.1/55.27 ± 6.01	Use of mobile phone app to guide patients in home cardiac exercise rehabilitation.	Usual care	➄
Frederix et al. (11)	Belgium	31/80	31(71-83)/77(71-83)	Patients use telemonitoring for daily self-assessment and transfer of data to the responsible telemedicine center.	Usual care	➀,➂
Koehler et al. (12)	Germany	31/73	31 ± 11/70 ± 10	Use of telemedicine platforms and wearable devices for self-monitoring and self-assessment of health status.	Usual care	➀–➂
Koehler et al. (13)	Germany	31/356	31.9 ± 10.8/66.9 ± 10.5	Use of telemedicine platforms and wearable devices for self-monitoring and self-assessment of health status.	Usual care	➀
Scherr et al. (14)	Australia	31/54	31(62-76)/66(62-72)	Using telemedicine systems for communication and wearable devices for self-monitoring (blood pressure, heart rate, weight).	Usual care	➀,➁
Athilingan et al. (15)	America	31/9	31.06 ± 4.02	Self-monitoring with mobile applications	Usual care	➅
Chen et al. (16)	China	31/260	31 ± 14/62 ± 15	Use cell phone text alerts and phone calls to remind patients.	Usual care and health education	➀–➂,➄
Dang et al. (17)	America	31/16	31.0 ± 9.4/60.3 ± 9.0	Use cell phone text alerts and phone calls to remind patients.	Usual care	➃–➅
Seto et al. (18)	Canada	31/50	31.1 ± 13.7/32.3 ± 13.7	Using a smartphone for remote monitoring of weight and blood pressure automatically sent to a cell phone *via* Bluetooth.	Usual care	➄
Melin et al. (19)	Sweden	31/40	31 ± 8	Using tablets to provide prognostic care knowledge, including medication guidance, lifestyle, health education, condition monitoring.	Usual care	➃,➅
Dendale et al. (20)	Belgium	31/80	31 ± 10	Use the Internet to monitor the condition, provide health education, diet guidance and exercise suggestions.	Usual care	➀–➂
Villani et al. (21)	Italy	31/40	31.3 ± 11.6/57.9 ± 11.9	Use remote monitoring technology for disease monitoring (such as heart rate, body weight, blood pressure, electrocardiogram).	Usual care	➀,➁
Vuorinen et al. (22)	Finland	31/47	31.3 ± 11.6/57.9 ± 11.9	Use mobile apps for disease monitoring and symptom self-assessment.	Usual care	➀,➂,➅
Cichosz et al. (23)	Denmark	31/154	31(59.5-77)/69(61-76)	Use of tablets and wearable devices to collect disease-specific data (blood pressure, pulse and weight), medication monitoring and dietary guidance, and rehabilitation training.	Usual care	➀,➃
Johnson et al. (24)	America	31/15	31.4 ± 23.4/60.7 ± 15.0	Use the Internet to provide educational videos and daily tips to patients.	Usual care	➀
Schmaderer et al. (25)	America	31/26	31.6 ± 14.2	Using mobile health apps to provide health education, self-monitoring to patients.	Usual care	➁
Völler et al. (26)	Germany	31/241	31.0 ± 11.5	Use the telemedicine platform motiva and wearable devices to provide prognosis care for patients, including measuring vital signs (blood pressure, heart rate and body weight).	Usual care and health diary	➀–➂
Yanicelli et al. (27)	Argentina	31/90	31.0 ± 10.6	Use the Home Remote Monitoring System application to collect collect weight, blood pressure, heart rate and list of symptoms (swollen ankles, swollen legs, shortness of breath, etc.) and provide health education to patients.	Usual care	➅
Piotrowicz et al. (28)	America	31/391	31.2 ± 10.0/62.1 ± 10.2	Patients underwent either an HCTR program (1 week in the hospital [initial stage] and 8 weeks at home; exercise training 5 times per week).	Usual care	➃
Saleh et al. (29)	Jordan	31/67	31(61-72)/65(62-72)	Tele group patients were asked to measure vital parameters (blood pressure, heart rate, body weight) on a daily basis at the same time.	Usual care	➃

➀All-cause mortality, ➁All-cause hospitalization rate, ➂Heart failure related hospitalization rate, ➃SF-23, ➄MLHFQ, ➅Self-care behavior.

### Primary outcomes

#### All-cause mortality

**Figure 4:**
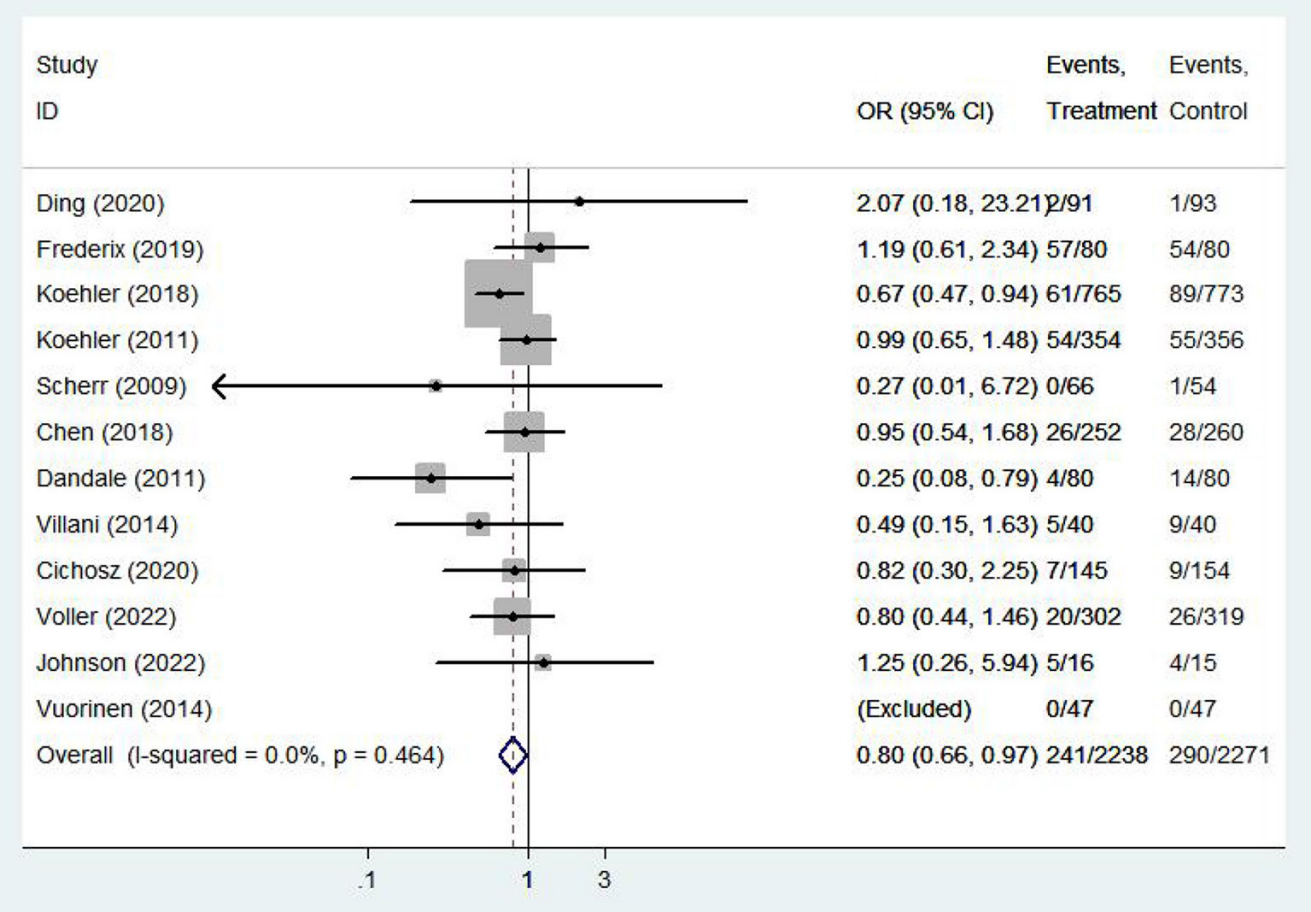
Forest plots showing the effects of all-cause mortality.

**Figure 5:**
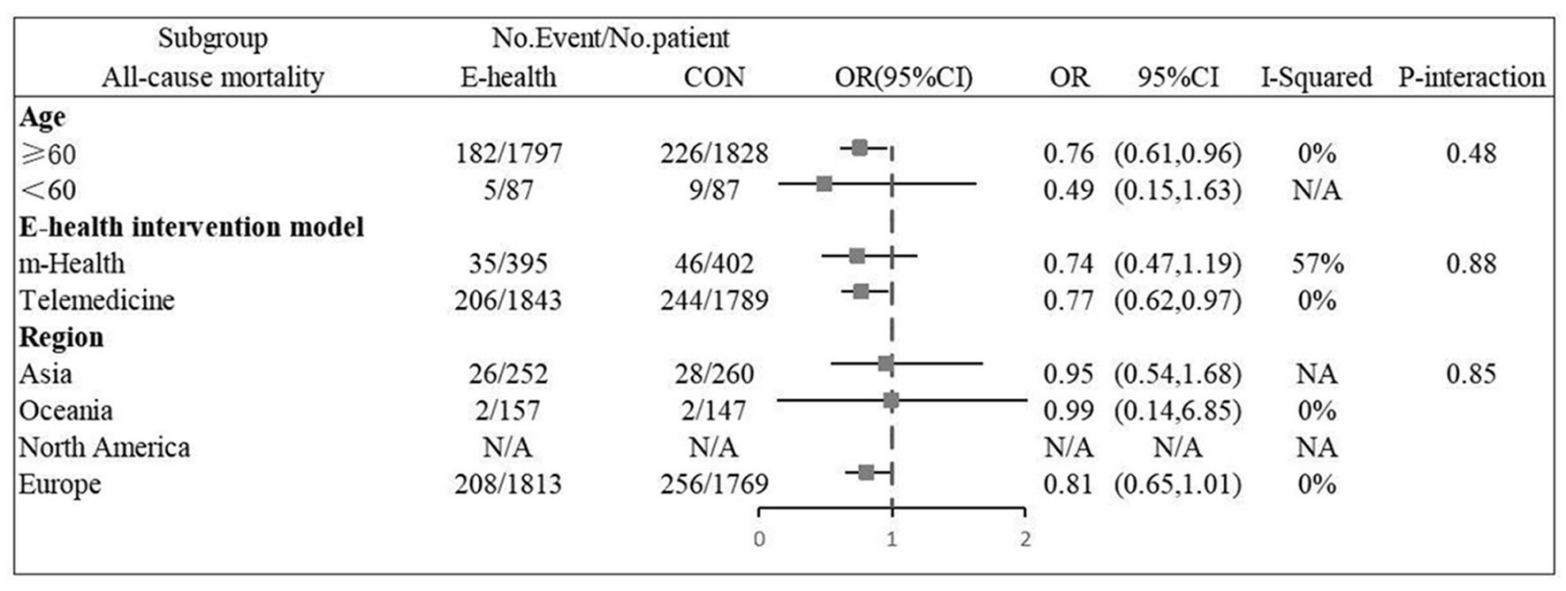
Subgroup analysis for outcome of all-cause mortality.

Twelve (9, 11–14, 16, 20–24, 26) studies involving a total of 4,059 patients assessed all-cause mortality. Due to the high homogeneity of the included studies (*I*^2^ = 0%, *P* = 0.46), the fixed effect model was used. There were statistically significant differences in the pooled odds ratios of all-cause mortality between patients treated with e-health and the control group (OR 0.801, 95% CI 0.65–0.98, *P* < 0.05), and meta-analysis showed that e-health interventions can significantly reduce all-cause mortality among patients (Figure 4). Subgroup analysis was performed based on the region of use, patient age, and the intervention model studied. There were no significant differences between the e-health intervention and usual care after subgrouping, as detailed Figure 5.

#### All-cause hospitalization rate

**Figure 6:**
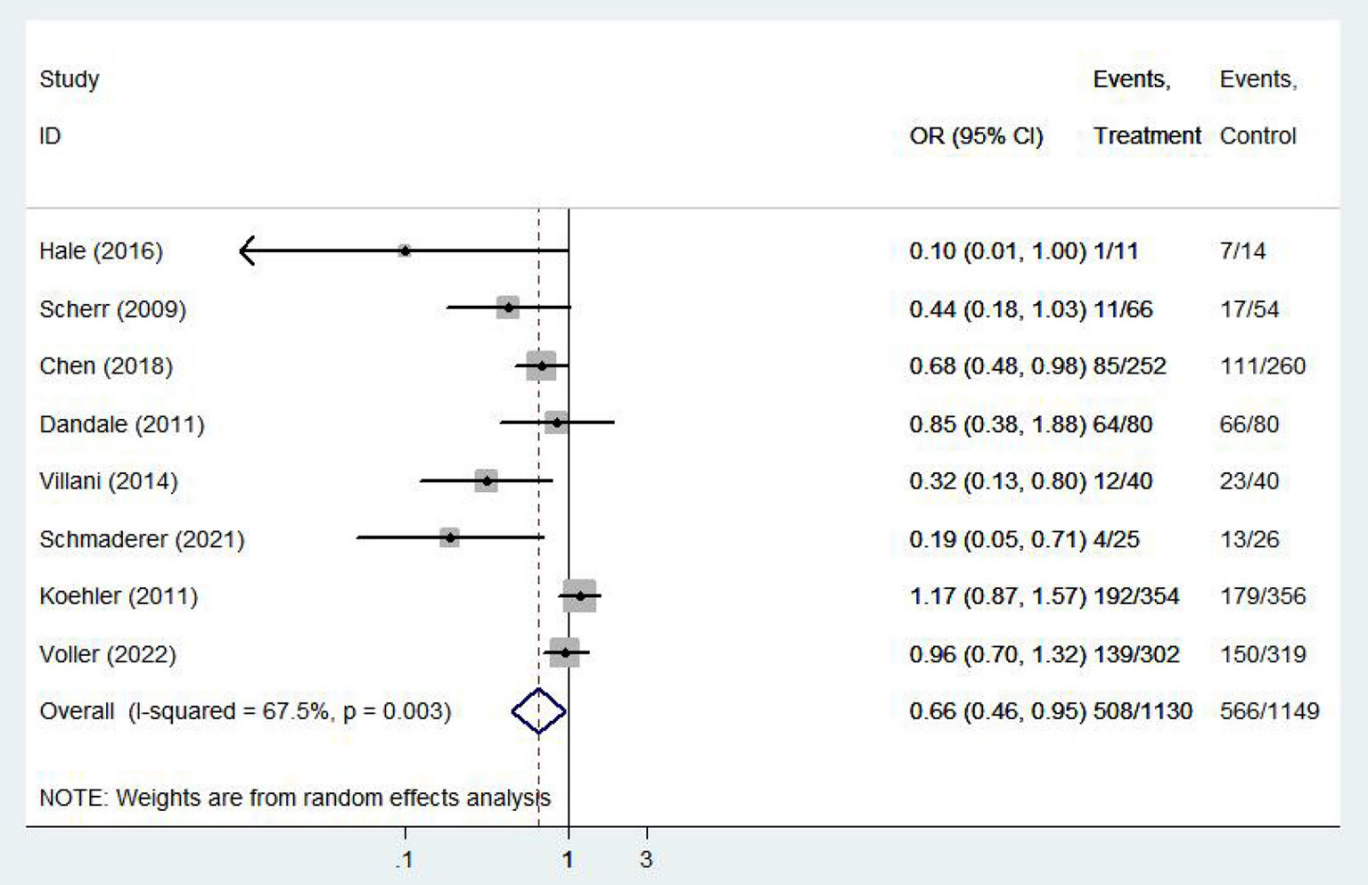
Forest plots showing the effects of all-cause hospitalization rate.

**Figure 7:**
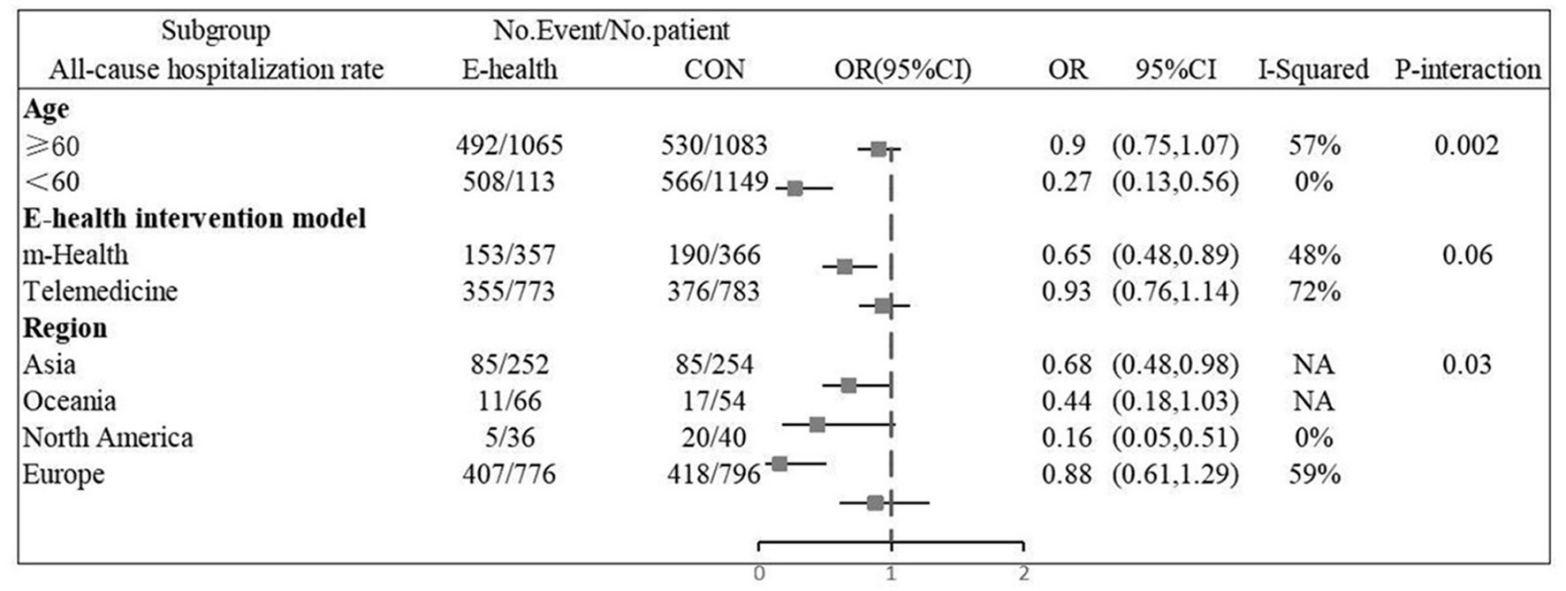
Subgroup analysis for outcome of all-cause hospitalization rate.

Eight (8, 13, 14, 16, 20, 21, 25, 26) studies involving 2279 patients reported findings on all-cause hospitalization rate. Due to the high heterogeneity, the random effects model was used in this study (*I*^2^ = 67.5%, *P* = 0.00). All-cause hospitalization rates were statistically significantly different between e-health patients and their control counterparts (OR 0.66, 95%CI 0.46–0.95, *P* < 0.05). According to our findings, e-health interventions can significantly improve all-cause mortality in patients with CHF (Figure 6). Sensitivity analysis using continuous omissions from individual studies had no significant effect on the overall combined OR, indicating that the combined OR was stable and valid. The subgroup analysis by population suggested that e-health intervention reduced all-cause hospitalization rates among under 60 years old CHF patients, but uncertainty exists for this outcome among over 60 years old patients. And subgroup analysis by region showed that the e-health intervention had a more significant effect on all-cause hospitalization rates in North American states. There was no significant difference between e-health intervention and usual care on all-cause mortality after the intervention model grouping, as detailed Figure 7.

#### Heart failure-related hospitalities

**Figure 8:**
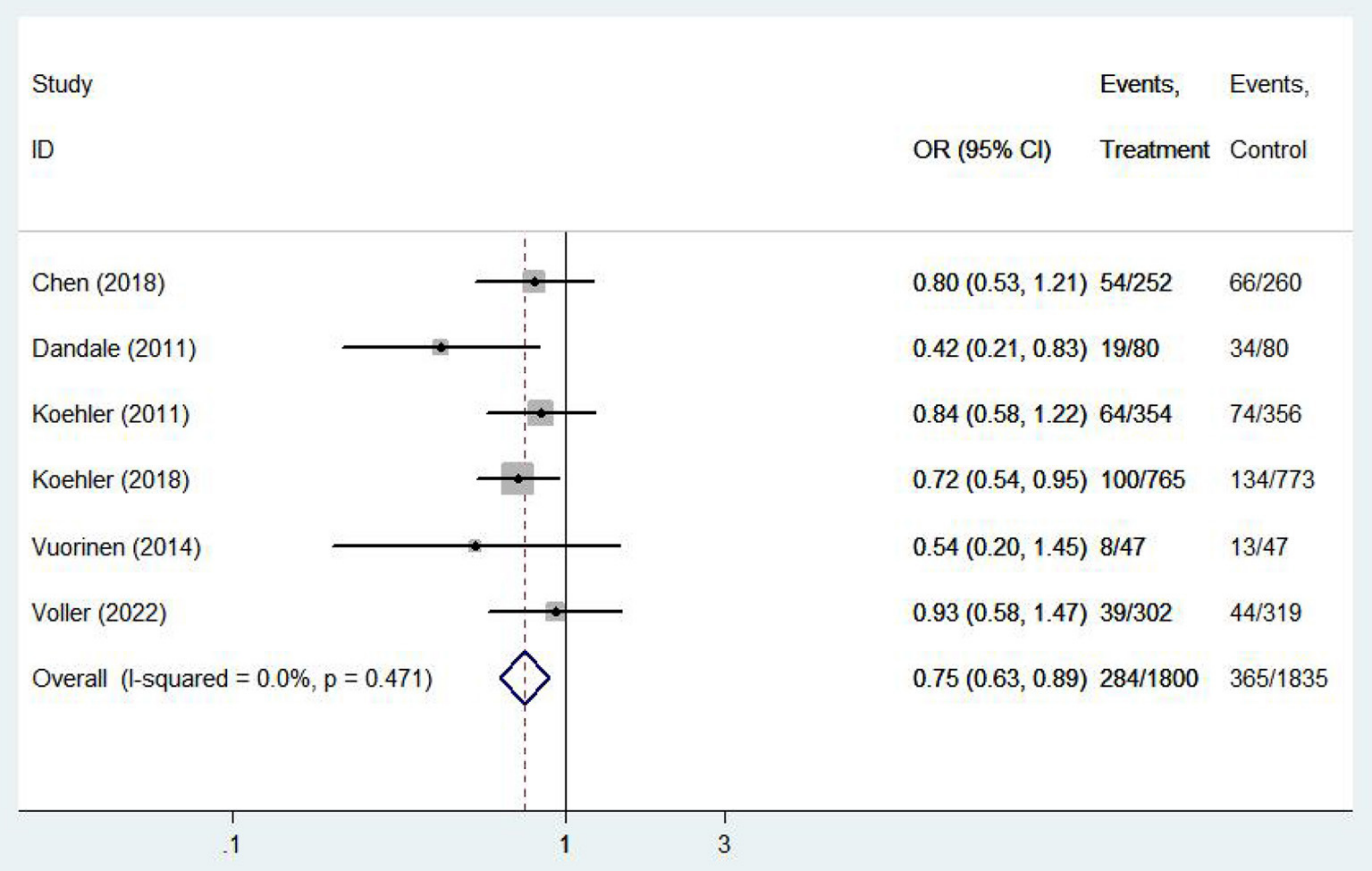
Forest plots showing the effects of heart failure related hospitalization rate.

**Figure 9:**
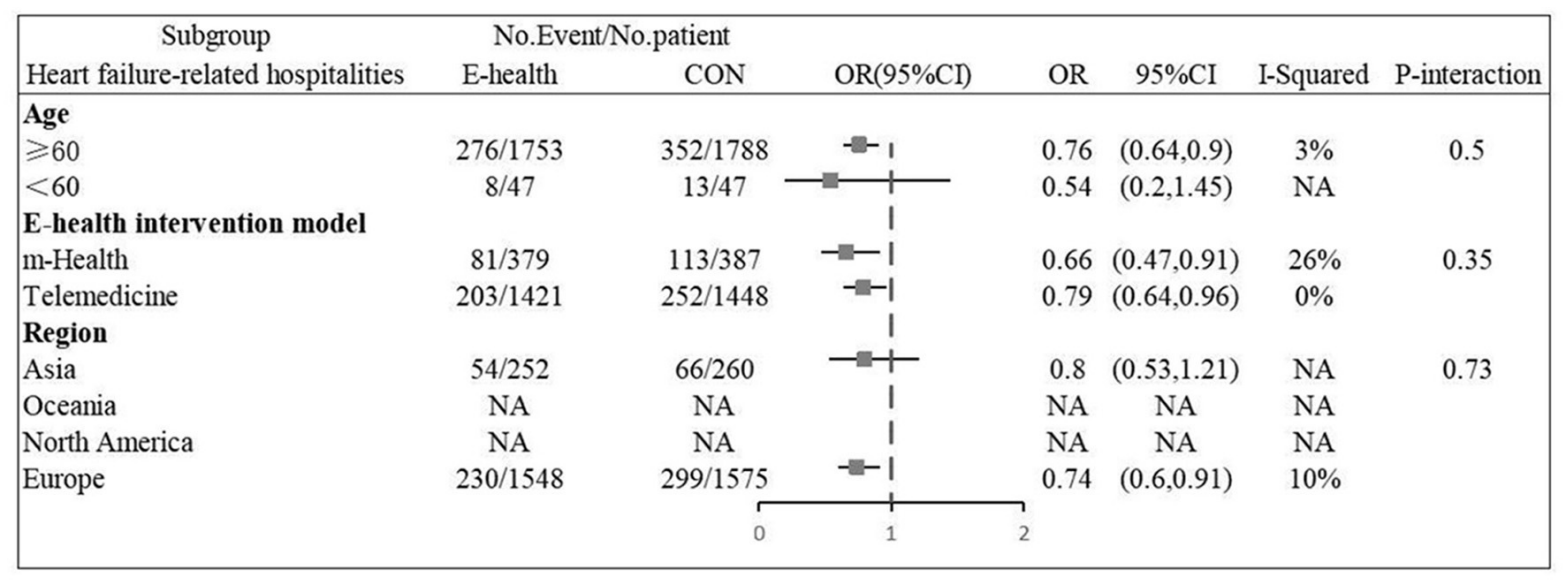
Subgroup analysis for outcome of heart failure related hospitalization rate.

Six (12, 13, 16, 20, 22, 26) studies involving 3,635 patients reported findings of heart failure-related hospitalization rate. Statistical heterogeneity was not present in these studies and therefore fixed effects model were used (*I*^2^ = 0.0%, *P* = 0.47). Meta-analysis showed that there was significant difference in heart failure-related hospitalization rate between e-health treatment group and the control group in pregnant women with hyperthyroidism (OR 0.750, 95%CI: 0.632–0.891, *P* < 0.01) (Figure 8). We found a reduced risk of heart failure-related hospitalizations in patients using the e-health intervention. Subgroup analysis showed no significant difference between the e-health intervention and usual care after grouping, as detailed Figure 9.

### The secondary outcome

#### Healthy life-related quality

**Figure 10:**
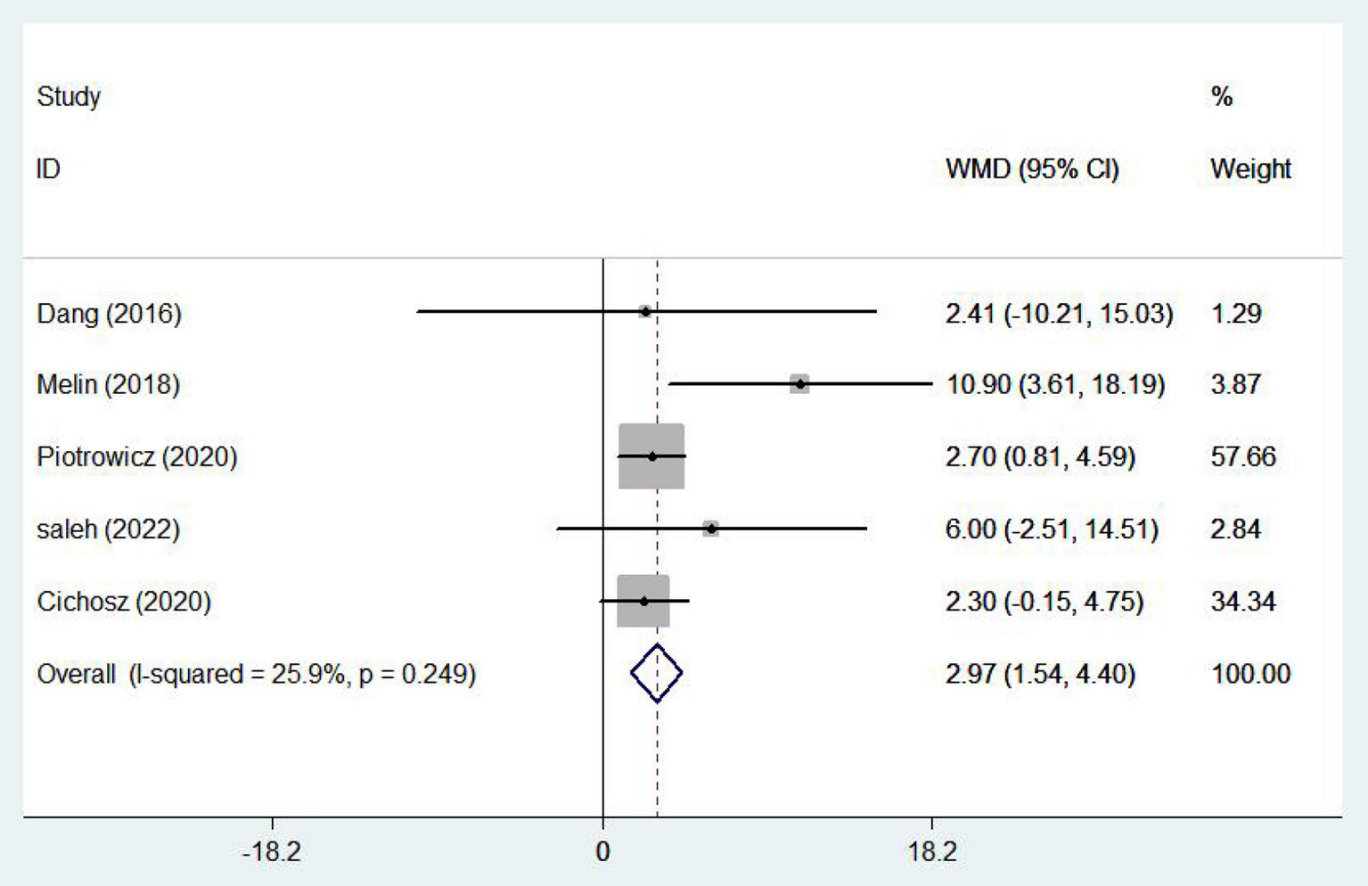
Forest plots showing the effects of SF-36.

**Figure 11:**
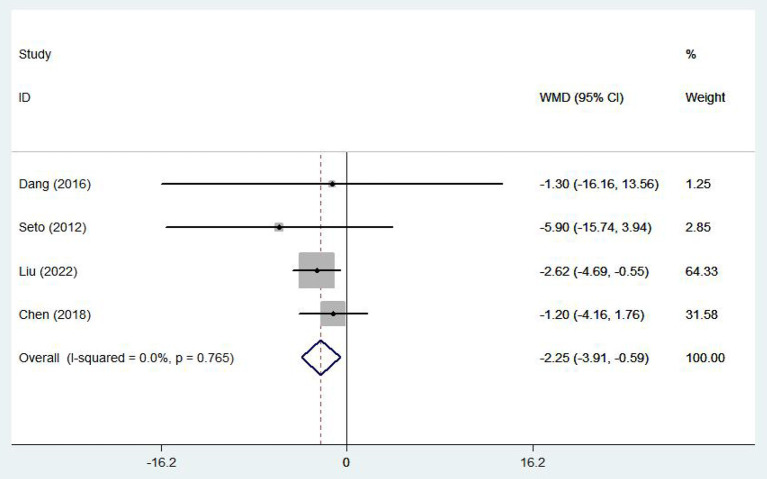
Forest plots showing the effects of MLHFQ.

Five (17, 19, 23, 28, 29) studies involving 1,337 patients reported healthy life-related quality using the SF-36 scale. There was no statistical heterogeneity among the studies (*I*^2^ = 25.9%, *P* = 0.249), so the fixed effect model was adopted. In patients with chronic heart failure, SF-36 scores were significantly higher, with the e-health treatment group outperforming the control group (WMD 2.97, 95%CI: 1.54 – 4.40, *P* < 0.05) (Figure 10). Four (10, 16–18) studies (704 patients) reported healthy life-related quality using the Minnesota Lifestyle Heart Failure Questionnaire (MLHFQ) and there was no statistical heterogeneity between studies (*I*^2^ = 0.0%, *P* = 0.765) so the fixed effect model was adopted. In the four individual trials using the MLHFQ, the results showed a significant difference between the e-health intervention and usual care (WMD −2.25, 95%CI: −3.91 – −0.59, *P* < 0.05) (Figure 11). According to the study, quality of life was improved in patients with e-health interventions.

#### Self-care behaviors

**Figure 12:**
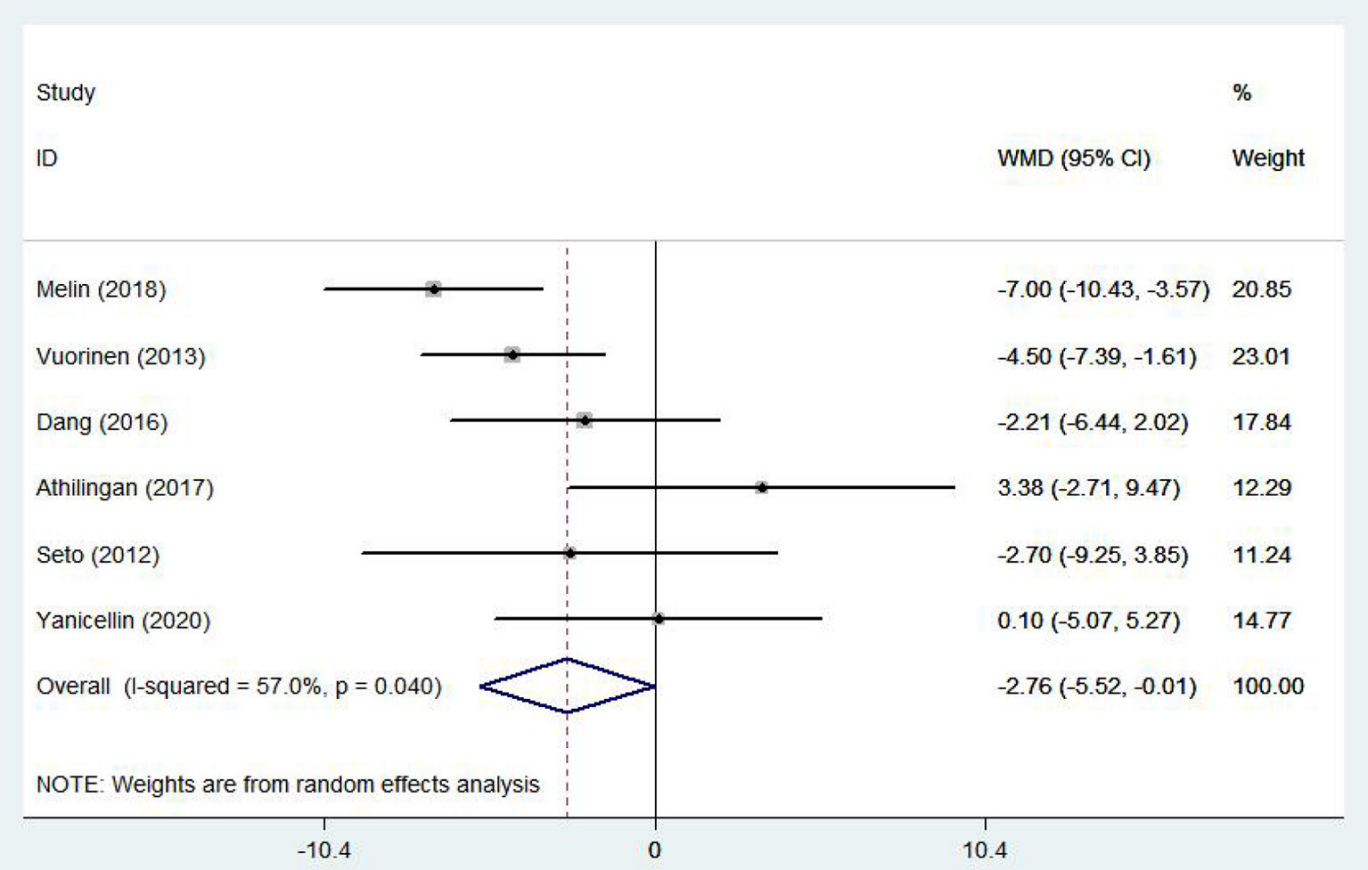
Forest plots showing the effects of self-care behaviors.

Six (15, 17–19, 22, 27) studies involving 386 patients reported findings on self-care behaviors. There was relatively large heterogeneity in the results (*I*^2^ = 57.0%, *P* = 0.04), so a random effects model was used. There were statistically significant differences in self-care behaviors between patients treated with e-health and the control group (WMD −2.76, 95%CI: −5.52−0.11, *P* < 0.05) (Figure 12).

#### Publication bias

**Figure 13:**
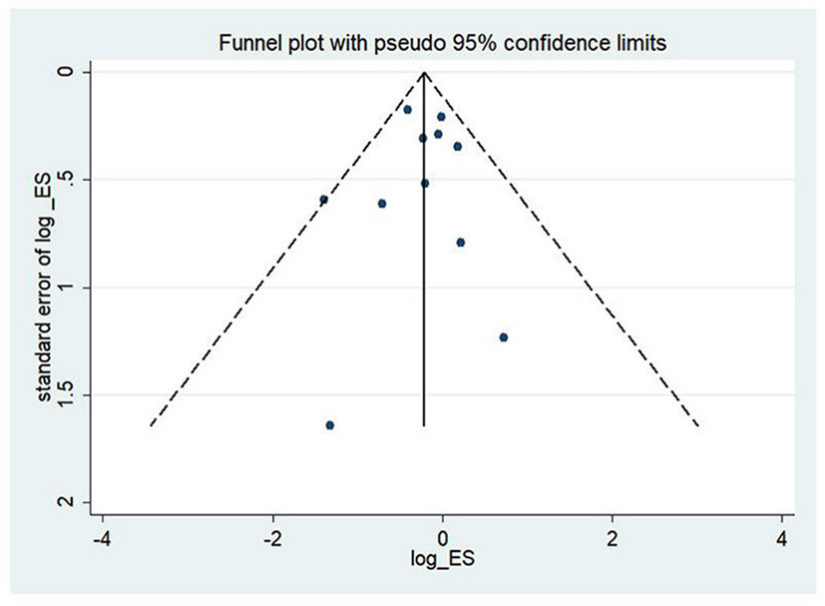
Funnel plot of all-cause mortality.

To assess the study's quality and bias risk, we used funnel plots and Begg's and Egger's tests. In the study of CHF patients, all-cause mortality showed a clear symmetric funnel plot (Figure 13). The results of Egger's (*P* = 0.77) and Begg's (*P* = 0.87) tests also confirmed that a relatively small study bias.

## Discussion

e-health interventions are based on the concept of mobile health care, where patient information data is uploaded to a monitoring platform or managed for patients with CHF *via* a home teleportation monitoring system, such as a wireless device or sensor worn on the patient to obtain vital signs, ECG and exercise data (30). In this Meta-analysis, the prognostic effect of e-health interventions on patients with CHF was brought up to date and the analysis included a total of twenty-two randomized controlled trials comparing the impact of e-health interventions (mobile devices and telemedicine care) with usual care on health outcomes in patients with CHF. Overall, e-health interventions were beneficial for all-cause mortality and quality of life in patients with CHF. Home-based electronic health interventions providing monitoring systems are highly effective in improving clinical outcomes, particularly in reducing all-cause mortality and all-cause hospitalization rates. Further subgroup analyses showed no significant differences between the two types of eHealth intervention models in all-cause mortality, all-cause hospitalization rates, and heart failure-related hospitalization rates.

Of the twelve studies that evaluated the relationship between e-health interventions and mortality, nine showed that e-health interventions significantly reduced all-cause mortality in patients with CHF, similar to the results of the meta-analysis by Kraijkamp et al. (31). e-health interventions can reduce all-cause mortality by providing real-time counseling and education to CHF patients and their families, monitoring daily patient weight, blood pressure and heart rate, fluid intake and observing edema in low hanging areas of the body, and by remote monitoring or professional guidance from health care professionals (32). Also, studies have shown significantly reduced all-cause mortality with more frequent vital sign measurements (more than twice a week). All-cause hospitalization rates and heart failure-related hospitalization rates are critical health economic indicators of patient discharge, transfer and post-discharge follow-up outcomes, and are helpful in assessing the prognosis of patients with CHF (33). The results of this study found that the e-health intervention group was able to significantly reduce all-cause hospitalization and heart failure-related hospitalization rates, with statistically significant differences. A subgroup analysis of all-cause hospitalization rates showed that the eHealth intervention was effective for CHF patients of all ages and regions. Almost all of the e-health interventions in this meta-analysis supported that monitoring heart failure-specific indicators (e.g., weight, blood pressure, and heart failure symptoms) may reduce heart failure-associated hospitalization rates. In addition, they can recognize exacerbations associated with other chronic diseases (e.g., chronic kidney disease or diabetes) or other types of cardiovascular disease, and electronic health interventions with remote monitoring and clinical feedback may provide alerts or draw attention to other acute diseases, which may reduce all-cause readmission rates for patients. e-health visits increased exponentially during the New Coronary Pneumonia pandemic. Current evidence supports the use of e-health interventions instead of post-discharge in-person visits. e-health interventions range in complexity from low to high and should be matched to the patient's risk profile. Regardless of the type of e-health intervention platform, it should be integrated with clinical practice to optimize healthcare delivery (34).

The report also examined the impact of e-health interventions on quality of life and self-management in patients with CHF. The current meta-analysis showed that patients with CHF who received e-health interventions had improved self-behavior management compared to usual care, which is consistent with the findings of Romano (35). Recent studies have found that telemedicine and mobile health interventions for patients with chronic heart failure also significantly improve self-management, medication adherence, and cognitive performance, as well as poor lifestyle and negative patient mood (36). In addition, e-health enables physicians to interact with patients by monitoring changes in their condition, offering immediate guidance, and collaborating with patients. All of these elements are consistent with the principles of self-management (37, 38). The positive effects of modern e-health could explain the immediate and short-term benefits of e-health based self-management behaviors in patients with chronic heart failure. Notably, there is inadequate evidence to determine whether these effects can be sustained in the medium or long term, and the quality of evidence obtained in the medium-term follow-up is low. Therefore, more clinical trials are needed to determine long-term effects.

## Limitations of the study

Statistical heterogeneity was excluded before pooling the data, but changes in the quality of intervention or routine care may introduce conceptual heterogeneity that cannot be fully explained in any meta-analysis. For example, e-health education interventions may differ in terms of goal setting, logbook use and other strategies that affect patient adherence or outcomes. Another limitation is that each type of intervention cannot account for the high or low quality of care provided.

## Conclusion

This meta-analysis included randomized controlled trials from different countries (China, USA, UK, Sweden, Australia, etc.) and suggests that e-health interventions may be applicable to populations in different countries and different healthcare systems. In conclusion, the results of this meta-analysis support the use of e-health interventions to improve the prognosis of patients with heart failure. e-health interventions reduce mortality, all-cause hospitalization and heart failure-related hospitalization rates, and improve quality of life in CHF patients.

## Author contributions

Conceptualization and writing—review and editing: XD and YX. Data curation: XD, WF, and YatW. Formal analysis: XD and YatW. Methodology: ZT, YatW, and GS. Supervision: YatW and ZT. Writing—original draft: XD and RG. All authors contributed to the article and approved the submitted version.

## References

[1] Van Nuys KE Xie Z Tysinger B Hlatky MA Goldman DP. Innovation in heart failure treatment: life expectancy, disability, and health disparities. JACC Heart failure. (2018) 6:401–9. 10.1016/j.jchf.2017.12.006 29525333 PMC5935537

[2] Ades PA Keteyian SJ Balady GJ Houston-Miller N Kitzman DW Mancini DM . Cardiac rehabilitation exercise and self-care for chronic heart failure. JACC Heart failure. (2013) 1:540–7. 10.1016/j.jchf.2013.09.002 24622007 PMC4271268

[3] Jaarsma T Hill L Bayes-Genis A La Rocca HB Castiello T Celutkiene J . Self-care of heart failure patients: practical management recommendations from the Heart Failure Association of the European Society of Cardiology. Eur J Heart Fail. (2021) 23:157–74. 10.1002/ejhf.2008 32945600 PMC8048442

[4] Chahal RS Chukwu CA Kalra PR Kalra PA. Heart failure and acute renal dysfunction in the cardiorenal syndrome. Clin. Med. (London, England). (2020) 20:146–50. 10.7861/clinmed.2019-0422 32188648 PMC7081827

[5] Borrelli B Ritterband LM. Special issue on eHealth and mHealth: Challenges and future directions for assessment, treatment, and dissemination. Health Psychol. (2015) 34s:1205–8. 10.1037/hea0000323 26651461

[6] Källander K Tibenderana JK Akpogheneta OJ Strachan DL Hill Z ten Asbroek AH . Mobile health (mHealth) approaches and lessons for increased performance and retention of community health workers in low- and middle-income countries: a review. J. Med. Internet Res. (2013) 15:e17. 10.2196/jmir.2130 23353680 PMC3636306

[7] da Fonseca MH Kovaleski F Picinin CT Pedroso B Rubbo P. E-health practices and technologies: a systematic review from 2014 to 2019. Healthcare (Basel, Switzerland). (2021) 9:1192. 10.3390/healthcare9091192 34574966 PMC8470487

[8] Hale TM Jethwani K Kandola MS Saldana F Kvedar JC. A Remote medication monitoring system for chronic heart failure patients to reduce readmissions: a two-arm randomized pilot study. J Med Internet Res. (2016) 18:e91. 10.2196/jmir.5256 27154462 PMC4890732

[9] Ding H Jayasena R Chen SH Maiorana A Dowling A Layland J . The effects of telemonitoring on patient compliance with self-management recommendations and outcomes of the innovative telemonitoring enhanced care program for chronic heart failure: randomized controlled trial. J Med Internet Res. (2020) 22:e17559. 10.2196/17559 32673222 PMC7381046

[10] Liu T Liu M. Effect of mobile internet technology in health management of heart failure patients guiding cardiac rehabilitation. J Healthc Eng. (2022) 2022:7118919. 10.1155/2022/7118919 35251573 PMC8894026

[11] Frederix I Vanderlinden L Verboven AS Welten M Wouters D De Keulenaer G . Long-term impact of a six-month telemedical care programme on mortality, heart failure readmissions and healthcare costs in patients with chronic heart failure. J Telemed Telecare. (2019) 25:286–93. 10.1177/1357633X18774632 29742959

[12] Koehler F Koehler K Deckwart O Prescher S Wegscheider K Kirwan BA . Efficacy of telemedical interventional management in patients with heart failure (TIM-HF2): a randomised, controlled, parallel-group, unmasked trial. Lancet (London, England). (2018) 392:1047–57. 10.1016/S0140-6736(18)31880-4 30153985

[13] Koehler F Winkler S Schieber M Sechtem U Stangl K Böhm M . Impact of remote telemedical management on mortality and hospitalizations in ambulatory patients with chronic heart failure: the telemedical interventional monitoring in heart failure study. Circulation. (2011) 123:1873–80. 10.1161/CIRCULATIONAHA.111.018473 21444883

[14] Scherr D Kastner P Kollmann A Hallas A Auer J Krappinger H . Effect of home-based telemonitoring using mobile phone technology on the outcome of heart failure patients after an episode of acute decompensation: randomized controlled trial. J Med Internet Res. (2009) 11:e34. 10.2196/jmir.1252 19687005 PMC2762855

[15] Athilingam P Jenkins B Johansson M Labrador M. A Mobile health intervention to improve self-care in patients with heart failure: pilot randomized control trial. JMIR cardio. (2017) 1:e3. 10.2196/cardio.7848 31758759 PMC6834206

[16] Chen C Li X Sun L Cao S Kang Y Hong L . Post-discharge short message service improves short-term clinical outcome and self-care behaviour in chronic heart failure. ESC heart failure. (2019) 6:164–73. 10.1002/ehf2.12380 30478888 PMC6352960

[17] Dang S Karanam C Gómez-Marín O. Outcomes of a mobile phone intervention for heart failure in a minority county hospital population. Telemed J E Health. (2017) 23:473–84. 10.1089/tmj.2016.0211 28051357

[18] Seto E Leonard KJ Cafazzo JA Barnsley J Masino C Ross HJ. Mobile phone-based telemonitoring for heart failure management: a randomized controlled trial. J Med Internet Res. (2012) 14:e31. 10.2196/jmir.1909 22356799 PMC3374537

[19] Melin M Hägglund E Ullman B Persson H Hagerman I. Effects of a tablet computer on self-care, quality of life, and knowledge: a randomized clinical trial. J Cardiovasc Nurs. (2018) 33:336–43. 10.1097/JCN.0000000000000462 29369123

[20] Dendale P De Keulenaer G Troisfontaines P Weytjens C Mullens W Elegeert I . Effect of a telemonitoring-facilitated collaboration between general practitioner and heart failure clinic on mortality and rehospitalization rates in severe heart failure: the TEMA-HF 1 (TElemonitoring in the MAnagement of Heart Failure) study. Eur J Heart Fail. (2012) 14:333–40. 10.1093/eurjhf/hfr144 22045925

[21] Villani A Malfatto G Compare A Della Rosa F Bellardita L Branzi G . Clinical and psychological telemonitoring and telecare of high risk heart failure patients. J Telemed Telecare. (2014) 20:468–75. 10.1177/1357633X14555644 25339632

[22] Vuorinen AL Leppänen J Kaijanranta H Kulju M Heliö T van Gils M . Use of home telemonitoring to support multidisciplinary care of heart failure patients in Finland: randomized controlled trial. J Med Internet Res. (2014) 16:e282. 10.2196/jmir.3651 25498992 PMC4275484

[23] Cichosz SL Udsen FW Hejlesen O. The impact of telehealth care on health-related quality of life of patients with heart failure: Results from the Danish TeleCare North heart failure trial. J Telemed Telecare. (2020) 26:452–61. 10.1177/1357633X19832713 30975047

[24] Johnson AE Routh S Taylor CN Leopold M Beatty K McNamara DM . Developing and implementing an mHealth heart failure self-care program to reduce readmissions: randomized controlled trial. JMIR cardio. (2022) 6:e33286. 10.2196/33286 35311679 PMC8981015

[25] Schmaderer MS Struwe L Loecker C Lier L Lundgren SW Wichman C . Mobile health self-management interventions for patients with heart failure: a pilot study. J Cardiovasc Nurs. (2021). 10.1097/JCN.0000000000000846 34369914 PMC10199463

[26] Völler H Bindl D Nagels K Hofmann R Vettorazzi E Wegscheider K . The first year of noninvasive remote telemonitoring in chronic heart failure is not cost saving but improves quality of life: the randomized controlled CardioBBEAT trial. Telemed J E Health. (2022). 10.1089/tmj.2022.0021 35325562 PMC9700331

[27] Yanicelli LM Goy CB González VDC Palacios GN Martínez EC Herrera MC. Non-invasive home telemonitoring system for heart failure patients: A randomized clinical trial. J Telemed Telecare. (2021) 27:553–61. 10.1177/1357633X19899261 31973633

[28] Piotrowicz E Mierzyńska A Banach M Jaworska I Pencina M Kowalik I . Quality of life in heart failure patients undergoing hybrid comprehensive telerehabilitation versus usual care—results of the Telerehabilitation in Heart Failure Patients (TELEREH-HF) Randomized Clinical Trial. Arch Med Sci. (2021) 17:1599–612. 10.5114/aoms.2020.98350 34900039 PMC8641512

[29] Saleh ZT Elshatarat RA Elhefnawy KA Helmi Elneblawi N Abu Raddaha AH Al-Za'areer MS . Effect of a home-based mobile health app intervention on physical activity levels in patients with heart failure: a randomized controlled trial. J Cardiovasc Nurs. (2022). 10.1097/JCN.0000000000000911 35389920

[30] Gokalp H de Folter J Verma V Fursse J Jones R Clarke M. Integrated telehealth and telecare for monitoring frail elderly with chronic disease. Telemed J E Health. (2018) 24:940–57. 10.1089/tmj.2017.0322 30129884 PMC6299847

[31] Kraaijkamp JJM van Dam van Isselt EF Persoon A Versluis A Chavannes NH Achterberg WP. eHealth in geriatric rehabilitation: systematic review of effectiveness, feasibility, and usability. J Med Intern Res. (2021) 23:e24015. 10.2196/24015 34420918 PMC8414304

[32] Guo X Gu X Jiang J Li H Duan R Zhang Y . A hospital-community-family-based telehealth program for patients with chronic heart failure: single-arm, prospective feasibility study. JMIR mHealth uHealth. (2019) 7:e13229. 10.2196/13229 31833835 PMC6935047

[33] Nair R Lak H Hasan S Gunasekaran D Babar A Gopalakrishna KV. Reducing All-cause 30-day hospital readmissions for patients presenting with acute heart failure exacerbations: a quality improvement initiative. Cureus. (2020) 12:e7420. 10.7759/cureus.7420 32351805 PMC7186095

[34] Lindenfeld J Albert NM Boehmer JP Collins SP Ezekowitz JA Givertz MM . HFSA 2010 comprehensive heart failure practice guideline. J Card Fail. (2010) 16:e1–194. 10.1016/j.cardfail.2010.04.004 20610207

[35] Ong MK Romano PS Edgington S Aronow HU Auerbach AD Black JT . Effectiveness of remote patient monitoring after discharge of hospitalized patients with heart failure: the better effectiveness after transition—heart failure (BEAT-HF) randomized clinical trial. JAMA Intern Med. (2016) 176:310–8. 10.1001/jamainternmed.2015.7712 26857383 PMC4827701

[36] Eisele M Harder M Rakebrandt A Boczor S Marx G Blozik E . Association of depression and anxiety with adherence in primary care patients with heart failure-cross-sectional results of the observational RECODE-HF cohort study. Fam Pract. (2020) 37:695–702. 10.1093/fampra/cmaa042 32358596

[37] Nolan RP Payne AY Ross H White M D'Antono B Chan S . An internet-based counseling intervention with email reminders that promotes self-care in adults with chronic heart failure: randomized controlled trial protocol. JMIR Res Protoc. (2014) 3:e5. 10.2196/resprot.2957 24480783 PMC3936276

[38] Son YJ Lee Y Lee HJ. Effectiveness of mobile phone-based interventions for improving health outcomes in patients with chronic heart failure: a systematic review and meta-analysis. Int J Environ Res Public Health. (2020) 17:1749. 10.3390/ijerph17051749 32156074 PMC7084843

