# Supplementary material for: Effect of e-health intervention on disease management in patients with chronic heart failure: A meta-analysis
Source: Front Cardiovasc Med. 2023 Feb 7;9:1053765. doi: 10.3389/fcvm.2022.1053765 (PMC9941331; doi:10.3389/fcvm.2022.1053765)
Supplement: Supplementary file 1 [file Data_Sheet_1.docx]

Supplementary material

Search strategy

Pubmed:672

#1(((((((e-Health) OR (eHealth)) OR (e-therapy)) OR (etherapy)) OR (m-health)) OR (mhealth)) OR (mobile health)) OR (teleHealth)；

#2(((Chronic heart failure) OR (chronic cardiac failure)) OR (Cardiac Failure)) OR (Congestive Heart Failure)

#2 AND #3

Web of Science:214

#1 AND #2

#1 TS= (((((((e-Health) OR (eHealth)) OR (e-therapy)) OR (etherapy)) OR (m-health)) OR (mhealth)) OR (mobile health)) OR (teleHealth)17869

#2 TS=(((Chronic heart failure) OR (chronic cardiac failure)) OR (Cardiac Failure)) OR (Congestive Heart Failure)9932

Scopus:42

#1 AND #2

#1 TITLE-ABS-KEY(((((((e-Health) OR (eHealth)) OR (e-therapy)) OR (etherapy)) OR (m-health)) OR (mhealth)) OR (mobile health)) OR (teleHealth) 697598

#2 TITLE-ABS-KEY(((Chronic heart failure) OR (chronic cardiac failure)) OR (Cardiac Failure)) OR (Congestive Heart Failure)4326

EBSCO:31

(S1 AND S2 )

#(((((((e-Health) OR (eHealth)) OR (e-therapy)) OR (etherapy)) OR (m-health)) OR (mhealth)) OR (mobile health)) OR (teleHealth) 36942

#2 (((Chronic heart failure) OR (chronic cardiac failure)) OR (Cardiac Failure)) OR (Congestive Heart Failure)54377

Cochrane:245

#1 AND #2

#(((((((e-Health) OR (eHealth)) OR (e-therapy)) OR (etherapy)) OR (m-health)) OR (mhealth)) OR (mobile health)) OR (teleHealth) in All Text - (Word variations have been searched) 2860

#2 (((Chronic heart failure) OR (chronic cardiac failure)) OR (Cardiac Failure)) OR (Congestive Heart Failure) in All Text - (Word variations have been searched) 438

Embase:462

#1 AND #2

#1 (((((((e-Health) OR (eHealth)) OR (e-therapy)) OR (etherapy)) OR (m-health)) OR (mhealth)) OR (mobile health)) OR (teleHealth) 104868

#2(((Chronic heart failure) OR (chronic cardiac failure)) OR (Cardiac Failure)) OR (Congestive Heart Failure)2341
